# 

**Published:** 1899-06

**Authors:** 


SUBSCRIPTION $1.00

PUBLISHED MONTHLY.

### ATLANTA JOURNAL-RECORD OF MEDICINE.

Successor to Atlanta Medical and Surgical Journal, Established 1855,
and Southern Medical Record, Established 1870.

Vol. I. Atlanta, Ga., June, 1899. No. 3.

